# Assessment of Psycho-Education of Carers Questionnaire: APEC-U” translation and cross cultural adaptation of an Urdu Version

**DOI:** 10.12669/pjms.35.4.661

**Published:** 2019

**Authors:** Imran Ijaz Haider, Farah Tiwana, Noor Zohra, Khaleeq Ur Rehman

**Affiliations:** 1 Prof. Dr. Imran Ijaz Haider, FRC Psych (London). Department of Psychiatry and Behavioral Sciences, Fatima Memorial Hospital College of Medicine and Dentistry, Lahore, Pakistan; 2 Ms. Farah Tiwana, MSc Mental Health Studies (UK). Department of Psychiatry and Behavioral Sciences, Fatima Memorial Hospital College of Medicine and Dentistry, Lahore, Pakistan; 3 Ms. Noor Zohra, PhD Scholar. Department of Human Development and Family Studies, College of Home Economics (University of the Punjab, Lahore, Pakistan); 4 Prof. Dr. Khaleeq Ur Rehman, FECSM (European Joint Committee). Department of Urology and Andrology, Fatima Memorial Hospital College of Medicine and Dentistry, Lahore, Pakistan

**Keywords:** Mental health, Psycho-education, Carers, Intraclass correlation, Translation

## Abstract

**Background and Objective:**

Psycho-education of carers is a part of good mental health practice. Our objective was to translate and validate the English questionnaire “Assessment of Psycho-Education of Carers” (APEC) into Urdu (APEC-U), for use in Pakistan.

**Methods:**

Following development and validation of APEC, it was translated into Urdu after consultation with experts and translators. After pretesting, one hundred and twenty bilingual male 67(55.8%) and female 53(44.2%) primary carers, who could understand both Urdu and English, and were carers for more than three months, were asked to fill in the self reporting Urdu questionnaire at the Fatima Memorial Hospital Psychiatry Out-Patient Department. The data were collected over a period of three months from September, 2018 to November, 2018.

**Main outcome measures:**

Responses were analyzed for internal consistency, reliability, Intraclass correlation coefficients and kappa statistics.

**Results:**

APEC-U was understandable and capable of assessing psycho-education in Urdu. High internal consistency was demonstrated on the full scale as 0.859. Degree of agreement (<0.001) between the Urdu and the originally developed English version was evaluated by Cohen’s Kappa, and a high degree of agreement was demonstrated.

**Conclusion:**

The Urdu questionnaire can adequately assess psycho-education of carers in psychiatric settings.

## INTRODUCTION

Psychoeducation is education of patients and families that includes information, knowledge and relapse prevention skills.^1^ It is an effective psychosocial treatment.^2^ Psycho-education is an essential element of effective mental health management, as recommended by the World Health Organization (WHO).^3^ To achieve its aim of educational empowerment, psycho-education should take place in the native language of the carer or the patient.^4^ For the Pakistani population, this means that psycho-education should ideally be delivered in the Urdu language.

Urdu is the national language as well as the language most commonly used and understood by the local population. Healthcare providers in government-run and private hospitals communicate with patients in Urdu and any research on the local population would require Urdu fluency. After successfully developing and validating APEC in English^5^, we recognized the need for an Urdu version. To this end, we have translated and validated our questionnaire in the Urdu language.

A number of local researchers have translated various questionnaires into Urdu. Notable examples include the translations of the Female Sexual Function Index^6^, the Quality of Life Scale^7^, the Beck Scale for Suicide Ideation^8^ and Diabetes Self-Management Questionnaire (DSMQ).^9^ However there were no available psycho-education assessment tools in Urdu for local use. The aim was to translate, linguistically adapt, and perform psychometric validation of the APEC into the Urdu language as “APEC-U”.

## METHODS

The translation of the originally developed English questionnaire into Urdu was done by following the steps of the method currently used by the American Association of Orthopedic Surgeons (AAOS) Outcomes Committee.^10^ Stage I: Initial translation: The APEC-U was reverse translated a few times to obtain a reliable translation. This was reviewed by a five member expert committee; a psychiatrist, an andro-urologist, a psychologist, a researcher and a linguistic expert for refinement. A committee of experts was provided with a list of the three domains and sub-domains and items allocated to these. They were requested to indicate their agreement to these statements which were then rephrased to ensure clarity. Stage II: Synthesis of the translations: In this step, two translated versions were made. Working with both translators, a common translation was formed. Problems were identified and any discrepancies were resolved between the forward translation and the developed original questionnaire. For experiential equivalence, the questionnaire items were replaced by similar items that were in accord with the culture of the local language. For conceptual equivalence, the committee examined the source and back translated version of the questionnaire for cultural equivalence in Pakistan. Stage III: Back translation: After finalizing version, a translator who was totally blind to the original questionnaire, translated it back into English. In this process of validity checking, it was ensured that the translated version reflected the same items as the original version. Stage IV: Expert committee: In this step, the expert committee consolidated all versions of the questionnaire and developed a pre-final version for pre testing. Semantic equivalence was done to check the meaning of the wordings of the translated questionnaire and to check for any grammatical discrepancies. For Idiomatic equivalence, the items which were difficult to understand were translated and easier wordings were found by the committee. Stage V: Test of the pre-final version: In this step, the translated version was then pre-tested on twenty participants including males 12(60%) and females 8(40%). Each respondent completed the self reporting questionnaire, and no difficulties were reported in understanding the translated version. Pretesting showed this translation to be suitable for participants. Stage VI: Submission of documentation to the developers or coordinating committee for appraisal of the adaptation process: In this step, the final testing of the questionnaire was done to ensure consistency between both versions.

The final Urdu version of APEC, herein referred to as APEC-U is the subject of discussion in this article. Institutional Review Board (IRB) approval of Fatima Memorial Hospital College of Medicine & Dentistry was received. The current study was undertaken during a period of three months from September, 2018 to November, 2018 at the department of Psychiatry and Behavioral Sciences Fatima Memorial Hospital College of Medicine and Dentistry. The participants were recruited by a purposive sampling technique. Oral and written informed consent was obtained from all one hundred and twenty participants and details of the study were shared with them.

### Demographic information of participants

According to the demographic information, most of the carers spent upto 8 hours 54(45%) with patients on a daily basis. According to the results, most of the carers 67(55.8%) were males and were husband of the patient 46(38%) and their source of information was internet 60(50%) and most of the participants 97(80.8%) reported that they needed more information about their patient’s illness. The mean value of the participant’s demographic information is presented in Table-I.

**Table I:** Demographic information of participants (n=120).

Demographics of Participants	Mean (SD)
Gender	1.44±0.49
Age	2.19±0.90
Education level	3.01±0.88
Time spent with the patient	5.75±2.37
Relationship to patient	3.28±1.19
Need for more information about patient’s illness	0.81±0.39
Source of information	3.58±1.04

Participants were carers of patients diagnosed with mental illnesses according to DSM 5 criteria.^11^ The inclusion criteria included bilingually educated (Urdu and English) males and females who were regular carers of patients since three months. They were recruited from the Psychiatry Out Patient Department. Participants were 18years and older in age, and were able to understand the language of the self-reported questionnaire.

After participants completed the self-reporting Urdu questionnaire, a washout period of one hour was observed. The questionnaire in the English language was given to each participant, and they were requested to complete it with respect to their patient as before. The responses on both the English and Urdu questionnaires were matched for agreement. Participants completed the self-reporting Urdu questionnaire and researchers verbally confirmed that the respondents understood their answers.

### Clinical evaluation of responses

Each respondent was again interviewed by the senior consultant psychiatrist, who was blind to their questionnaire responses. This was done to ensure their understanding of their relative’s disorder, and was matched with their responses to the two versions of the questionnaire. Participants responded to four point likert-scale options ranging from “not aware” (1) to “fully aware” (4). The original APEC was developed in the English language and has now been psychometrically translated into the Urdu language by the forward and back translation method. It was translated into Urdu to allow for a broader range of applications.

### Statistical Analysis

Mean values of total and domain scores of the APEC-U as well as their cronbach’s α coefficients were determined to assess its internal consistency and reliability. Agreement between responses of the same participant to the original English and the Urdu version(APEC-U) were analyzed using Kappa statistics using the Statistical Package for the Social Sciences (SPSS) version 21 (SPSS, Inc., Chicago, IL, USA).

## RESULTS

Cronbach’s α coefficients were determined for total and domain scores of the questionnaire, which were significantly high, ranging from 0.823 to 0.859 for the entire sample of one hundred and twenty participants. This indicates that the questionnaire has good internal consistency and reliability. Individual domain scores and full-scale scores were calculated by adding the three domain and sub domain scores as presented in Table-II.

**Table II:** Mean values and cronbach’s α coefficient of the questionnaire for assessment of psychoeducation of carers questionnaire- Urdu (APEC-U) (n=120).

Domain name	Mean ± SD	Cronbach’s alpha	Number of items
Domain1: About nature of the illness	3.011±0.823	0.823	3
a. Name of illness	3.208±1.107		
b. Common signs and symptoms	3.167±0.823		
c. Progress of the illness	2.658±0.921		
Domain 2: Satisfaction & benefit of information provided	3.103±0.748	0.859	3
e. Was information provided Understandable?	3.225±0.835		
f. Satisfaction about information Provided	3.067±0.886		
g. Was information provided was helpful	3.016±0.819		
Domain 3a: Information regarding use of medications	3.32±0.683	0.845	3
k. Information about use of medicines prescribed	3.358±0.754		
l. Information how many times this medication is to be taken	3.400±0.738		
m. Satisfaction about the information regarding use of medications	3.208±0.849		
Domain3b: Information regarding side-effects of medications	2.21± 0.76	0.844	3
n. Information about the side effects of medications prescribed	2.275±0.916		
o. Information what to do in case of side effects of medications	2.108±0.838		
p. Satisfaction about the information provided regarding the side effects of the medications	2.250±0.852		
Full scale scores	34.94±6.503	0.859	12

†The full-scale score is calculated by adding the three domain and sub domains scores.

SD = standard deviation.

The degree of agreement between the Urdu version and the originally English version was evaluated by Cohen’s Kappa, and a high degree of agreement was demonstrated as shown in Table-III.

**Table III:** Kappa values of APEC-E and APEC-U.

Statements		Kappa value APEC-E & APEC-U
Domain 1: Nature of the illness		
a	Name of illness	1.000
b	Common signs and symptoms	0.987
c	Progress of the illness	1.000
Domain 2: Satisfaction & benefit of information provided by the mental health professional		
d	Was information provided understandable	0.987
e	Satisfaction about information provided	0.797
f	Was information provided was helpful	0.835
Domain 3a: Information regarding medications use		
g	Information about use of medicines prescribed	0.821
h	Information how many times this medication is to be taken	0.801
i	Confident about the information regarding use of medications	0.909
Domain 3b: Information regarding side-effects of medications		
j	Information about the side effects of medications prescribed	0.826
k	Management in case of side effects	0.798
l	Confident about the information provided regarding the side effects of the medications	0.869

*The p-value for all the kappa statistics were significant with p-value <0.001

The time taken to fill in the Urdu version was less than the time taken to complete the English version as shown in Table-IV.

**Table IV:** Time comparison of APEC-E and APEC-U.

APEC version	Mean (time)	SD (time)
APEC-English	5minutes 37 seconds	1 minutes 16 seconds
APEC-Urdu	5minutes 32seconds	0minutes 52seconds

## DISCUSSION

Our objective was to translate the originally developed questionnaire APEC. A common challenge is the development of a comprehensive translated version for an average person that is culturally appropriate, and close to the original. Once this objective has been achieved, we will aim for systematic testing and translation into various regional languages. Researchers and clinicians must have access to reliable measures of their interest in their local language to conduct cross-cultural research and provide quality patient care^12^. The present study has been undertaken in a systematic way to develop a translation of the original English version of APEC and to validate its responses for comprehensiveness and psychometric acceptability. To date, we have no validated instrument in Urdu to assess whether psycho-education is being effectively provided. The main results of our study showed that APEC-U is a valid and reliable self-reported instrument to assess psycho-education of carers in our population.

We have generated a psychometrically valid Urdu version of APEC that is linguistically equivalent to the original English version. The values reflected by an instrument and the meanings of its component constructs may vary from one culture to another.^13^ APEC-U allows mental health professionals to enhance psycho-education according to cultural values and meanings. Data generated through APEC-U were comparable with the data generated by the original English version. We hope that APEC-U (available online as a supplementary file) will encourage mental health professionals to address the needs of carers in their clinical practice. This questionnaire will not only encourage mental health professionals and researchers to provide and evaluate psycho-education; it will also encourage carers to take an active role in their patient’s treatment and recovery. Researchers setting out to conduct research employing questionnaires in non-English speaking populations need instruments that have been validated in the original nativelanguages.^14^ It is very important to emphasize that our objective is to translate our originally developed English questionnaire APEC into the local language to facilitate clinicians, carers and researchers. Our results show a good amount of correlation and internal consistency between the English and Urdu versions of APEC. According to one research article, two very important aspects of the translation method are first, the factors that affect the quality of the translated version and second, equivalence between the original and the targeted version^15^. We believe these have been covered by our current translation.

### Limitation of this questionnaire

It is its sole focus on the psycho-education of carers and literate population. This excludes patients and others involved. Moreover, those who were unable to understand Urdu language and were illiterate were excluded from the study, and thus this limits the reach of APEC-U. However, it provides a comprehensive measure of the domains of psycho-education and can easily be used for research on literate Urdu population.

In Pakistan, the burden of care is on the eldest member of the family or nearest blood relative. Therefore, in our culture families are given ultimate authority and are responsible to take decision on behalf of patient.^16^ Therefore, this makes it very important for carers to receive psycho-education, as it is known to ease burden of care and it will help in treatment compliance. A point of interest in this study was that as Urdu is the local language, therefore it took less time for participants to fill in the Urdu version of the questionnaire as compared to the English version. The scoring appendix of APEC-U is similar to the original questionnaire. The lowest to highest score ranges from 12-48.

## CONCLUSION

This translation has established that APEC-U is a psychometrically valid instrument to evaluate the multi-faceted psycho-educational needs of carers of patients with mental illnesses. It has also been shown to be equivalent to the original English version of APEC, and can hence be used for research in our region.
